# Cooled Radiofrequency Ablation in Shoulder Pain: A Cohort study

**DOI:** 10.7759/cureus.79922

**Published:** 2025-03-02

**Authors:** Teresa Nava-Obregon, Dionisio Palacio-Ríos, Francisco López-Ríos, Sandra Castillo-Guzmán, Juan Francisco Torres-Pérez, Mario Simental-Mendía, Carlos Acosta-Olivo

**Affiliations:** 1 Pain and Palliative Care Clinic, Anesthesiology Service, Hospital Universitario “Dr. José E. González” Universidad Autónoma de Nuevo León, Monterrey, MEX; 2 Anesthesiology Service, Hospital Universitario “Dr. José E. González” Universidad Autónoma de Nuevo León, Monterrey, MEX; 3 Geriatric Clinic Service, Hospital Universitario “Dr. José E. González” Universidad Autónoma de Nuevo León, Monterrey, MEX; 4 Orthopedics and Trauma Service, Hospital Universitario “Dr. José E. González” Universidad Autónoma de Nuevo León, Monterrey, MEX; 5 Orthopedics and Trauma Service, School of Medicine, Hospital Universitario “Dr. José E. González” Universidad Autónoma de Nuevo León, Monterrey, MEX

**Keywords:** cooled radiofrequency ablation, pain management, sensitive shoulder innervation, shoulder function, shoulder pain

## Abstract

Objective: This study aims to evaluate the pain and clinical evolution of patients with shoulder pain with cooled radiofrequency ablation (CRFA).

Methodology: A cohort prospective study of patients with shoulder pain for >3 months. All patients included were treated with CRFA in the sensitive innervation around the shoulder (lateral pectoral, suprascapular nerve, axillary nerve), and were followed for 24 weeks. Pain was evaluated with the visual analog scale, while clinical evolution was evaluated with several scales of shoulder function.

Results: Fifteen patients were included. All patients improved significantly the pain during the time of the study. The clinical function of the shoulder shows a significant improvement during the 24 weeks of follow-up.

Conclusions: CRFA is a medical procedure that helps to improve pain and function related to shoulder pain independently of the primary pathology in the shoulder.

## Introduction

Shoulder pain is a common complaint in the general population, with a lifetime prevalence estimated at 7-67%, increasing with age [1]. The incidence of shoulder pain is estimated to be 0.9%-2.5% across all age groups [1]. Various orthopedic conditions unrelated to acute trauma, such as rotator cuff disease, adhesive capsulitis, labral injuries, osteoarthritis (OA), and cuff arthropathy, are associated with chronic shoulder pain, particularly in patients over 45 years old [2]. Numerous non-surgical treatment options are available, including nonsteroidal anti-inflammatory drugs (NSAIDs), lifestyle modifications, patient education, physiotherapy, and corticosteroid injections [2].

Radiofrequency ablation (RFA) is another option for managing intractable shoulder pain. Several types of thermal ablative procedures exist, including pulsed RFA (PRFA) at 42 ºC, conventional RFA, cooled RFA (CRFA), and cryoneurolysis. These procedures use thermal energy to affect the nerves responsible for pain modulation and transmission [3]. The lesion size created by monopolar RFA (MRFA) depends on various procedure-related factors, such as cannula diameter, active tip length, temperature, and application time. A larger cannula diameter, heating lateral and distal to the active tip, higher tip temperature, and longer application duration result in a more extensive lesion [4].

In standard RFA, the temperature reached around the probe and surrounding tissue is approximately 80 ºC. However, a key limitation of this treatment is tissue charring at the electrode interface [5,6]. This charring acts as an insulator, preventing further energy transmission and thereby limiting lesion size. Typically, RFA lesions are described as elliptical [7]. RFA has been effectively used to create controlled lesions on the suprascapular nerve in patients with shoulder pain, demonstrating improvements in pain relief and range of motion [8].

CRFA was developed to minimize the charring and insulation issues associated with RFA. By circulating cooled water through the probe tip, CRFA maintains lower interface temperatures, around 60 ºC [7,9]. This technique allows for more energy delivery to the surrounding tissues, resulting in a larger heat lesion. The more extensive, spherical lesion shape created by CRFA facilitates broader denervation, reducing the likelihood of missing the target nerve [7].

Local neuronal denervation achieved with CRFA is greater than that of traditional RFA [9,10]. A study on patients with glenohumeral OA treated with CRFA reported significant pain reduction and functional improvement over a six-month follow-up period [11]. Several functional scales, such as the Shoulder Pain and Disability Index (SPADI) and Disabilities of the Arm, Shoulder, and Hand (DASH), are commonly used to evaluate therapeutic outcomes in shoulder dysfunction [12]. The University of California at Los Angeles Shoulder Score (UCLASS) is an effective tool for assessing quality of life following shoulder surgery [13]. These scales assess pain severity, functionality, range of motion, and patient satisfaction.

The primary objective of this study was to evaluate pain reduction, and the secondary outcomes were clinical scales and adverse effects in patients with shoulder pain of various etiologies treated with CRFA.

## Materials and methods

Study design and patients

This prospective cohort study was approved by our institution’s Ethics Committee. All participants provided informed consent. The inclusion criteria were patients with shoulder pain persisting for ≥3 months without improvement despite pharmacological treatment or rehabilitation, pain intensity >50% on the visual analog scale (VAS), a stable analgesic medication dosage for at least 30 days, a confirmed diagnosis of OA, chronic painful shoulder syndrome, or adhesive capsulitis, pain relief following local anesthetic application over the joint’s sensory branches, normal coagulation parameters, and a 50% pain reduction (VAS) following a fluoroscopy-guided suprascapular nerve block with 2 mL of 1% lidocaine [14]. The exclusion criteria were acute shoulder pain relieved with oral analgesics, infection at the treatment site, psychiatric disorders, opioid treatment exceeding 90 mg/day of morphine equivalents, prior shoulder surgery, and pregnancy. Patients who withdrew from treatment or did not complete follow-up evaluations were eliminated.

Procedure

The diagnostic block followed the protocol described by Tran et al. [14], while the CRFA technique adhered to the original description by Eckmann et al. [15]. A cooled radiofrequency (RF) cannula was used (CRK 17-75-2 Coolief Cooled Radiofrequency Kit Advanced, Avanos Medical, New Jersey) along with a Halyard Coolief Cooled RF Pain Management Generator PMG Model 4 Advanced (Halyard Health, Alpharetta, GA).

CRFA Procedure

Suprascapular nerve approach: With the patient in the prone position, fluoroscopic guidance was used with a 15º lateral and 15º caudal tilt to visualize the glenoid fossa medial to the humeral head. The target was lateral and posterior to the scapula, just before the glenoid fossa. The RF cannula was introduced laterally in tunnel vision, inferior to the spinoglenoid notch. Sensory and motor tests were conducted. CRFA was applied at 60 ºC for 2 minutes and 30 seconds, twice.

Lateral pectoral nerve approach: The patient was supine. A fluoroscopic AP shoulder image was obtained, tilted 15º laterally and 15º cranially toward the coracoid process. The CRFA cannula (CRK 17-75-2) was introduced through a 2-cm deep vision tunnel. Sensory and motor tests were conducted. CRFA was applied at 60ºC for 2 minutes and 30 seconds, twice.

Axillary nerve approach: The patient was supine. Fluoroscopy was used to identify the inferior and lateral edge of the major tuberosity. The CRFA cannula was inserted under tunnel vision to a depth of 4-7 cm. Sensory and motor tests were performed. CRFA was applied at 60 ºC for 2 minutes and 30 seconds, twice.

After ablation, 1 mL of 2% ropivacaine and 1 mL of 4 mg/mL dexamethasone were administered at each treated nerve site.

All patients were managed post-procedure with 750 mg of acetaminophen every eight hours for three days, along with the application of ice to the treatment area for 20 minutes every six hours for two days.

Follow-up and outcome measures

The primary outcome was evaluating the pain using the VAS, while the secondary outcome was evaluating the functionality using the VAS, SPADI, UCLA, and DASH scales, and adverse events. All patients were evaluated at baseline and weeks 1, 4, 12, and 24 post-treatment.

Statistical analysis and sample size calculation

Descriptive statistics were calculated for quantitative variables (median, interquartile range) and categorical variables (frequencies, percentages). Repeated measures ANOVA was used to compare clinical scale scores over time. Post hoc analysis was conducted to compare baseline evaluations with follow-up measurements. Statistical significance was set at *P* < 0.05. The software used was R-4.3.2 for Windows (R Foundation for Statistical Computing, Vienna, Austria).

The sample size calculation was performed using the formula for estimating sample size in studies with paired measurements, considering the difference in means before and after treatment. The following equation was used: *n* = ((*Zα*/2 + *Zβ*) × *σd*/*Δ*)2, where *Zα*/2 = 1.96 corresponded to a 95% confidence level, *Zβ* = 0.84 represented the critical value associated with an 80% statistical power, *σd* = 4 was the estimated standard deviation of the pre-post differences, and *Δ* = 3 was the expected mean difference. Considering the above, a sample size of 14 subjects was obtained.

## Results

The study included 15 patients, with a median age of 64 years (interquartile range [IQR] 52-78). Most participants were women (*n *= 12, 80%). Most patients (*n *= 10) presented with painful shoulder syndrome unrelated to joint disease. The most common comorbidity was rheumatoid arthritis. Additional demographic data are presented in Table 1.

**Table 1:** Demographic characteristics of the included patients.

Demographic characteristics		Values, *n* (%)
Gender	Female	12 (80%)
	Male	3 (20%)
Occupation	Housewife	8 (53.3%)
	Employed	4 (26.5%)
	Retired	3 (20%)
Diagnostic	Rotator cuff tendinopathy	9 (60%)
	Glenohumeral arthritis	5 (33.3%)
	Adhesive capsulitis	1 (6.6%)
Co-morbidities	Rheumatoid arthritis	8 (53.3%)
	Systemic arterial hypertension	7 (46.6%)
	Diabetes mellitus	7 (46.6%)
	Cardiopathies	13 (86.6%)
	Hypothyroidism	2 (13.3%)

VAS

**Figure 1:**
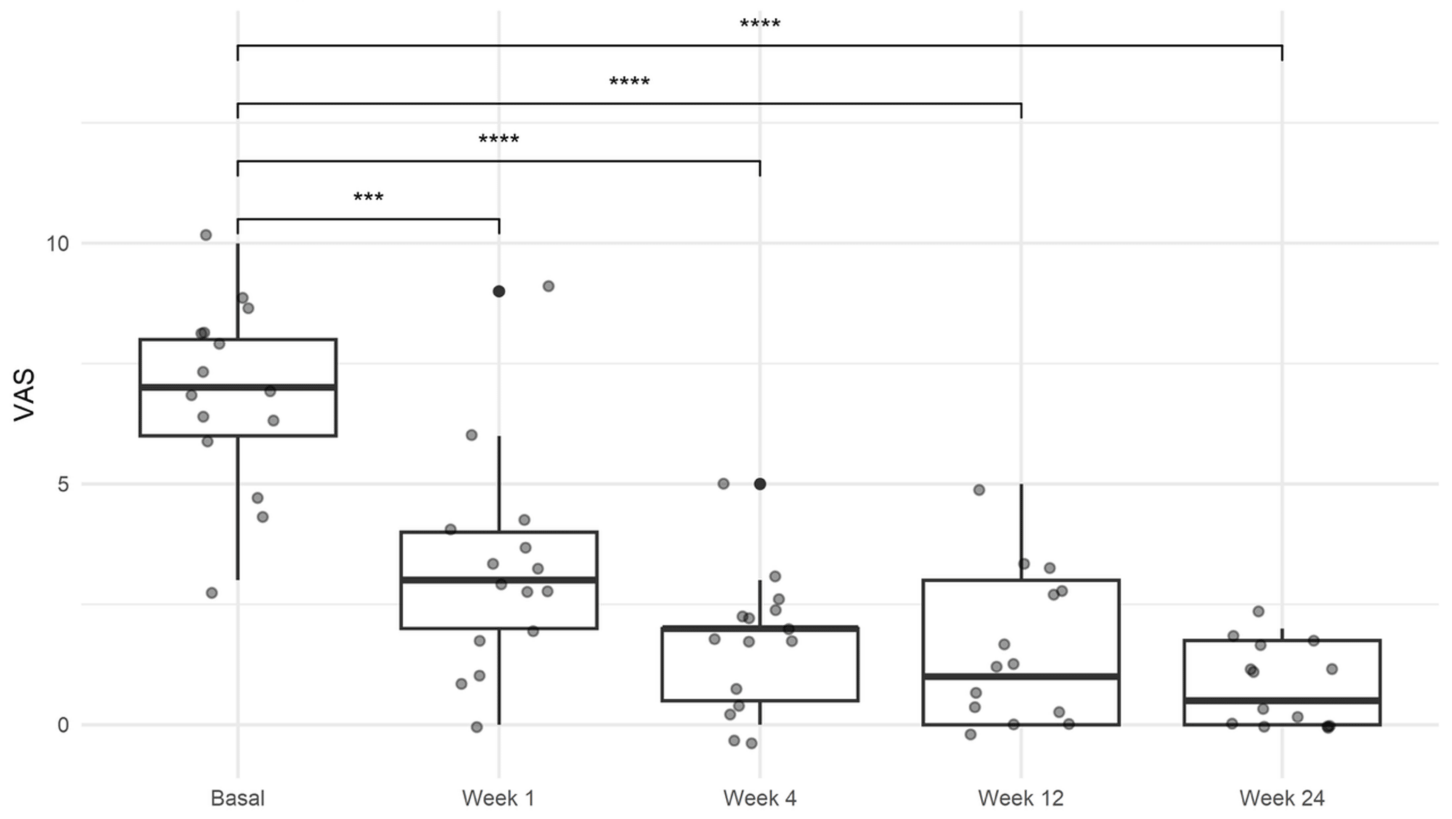
Results of the visual analog scale (VAS) for pain. A significant improvement was observed during the follow-up of the patients. One-way ANOVA with Tukey’s multiple comparison post hoc test. ***P < 0.001. ****P < 0.0001. ANOVA, analysis of variance

The median pain score at baseline was 7.0 (IQR 6.0-8.0). Following treatment administration at the first visit, patients exhibited a significant reduction in pain sustained throughout the follow-up period. At the final evaluation, the median pain score was 0.5 (IQR 0.0-1.7; *P *< 0.001; Figure 1; Table 2).

**Table 2:** Values of the pain and clinical scales during the follow-up. *P *< 0.005 was considered as significant. M, median; IQR, interquartile range; VAS, visual analogue scale; SPADI, Shoulder Pain Disability Index; DASH, Disabilities of the Arm, Shoulder, and Hand; ANOVA, analysis of variance; UCLA, University of California at Los Angeles

Score	M (IQR)					ANOVA, *P*-value
	Basal	Week 1	Week 4	Week 12	Week 24	
VAS	7.0 (6.0-8.0)	3 (2 - 4)	2.0 (0.5-2.0)	1.0 (0.0-3.0)	0.5 (0.0-1.7)	<0.001
SPADI	76.1 (65.1-78.2)	29.9 (19.1-38.1)	20.7 (17.3-25.7)	12.4 (8.1-20.1)	6.3 (1.8 - 11.7)	<0.001
UCLA	7.7 (6.7-9.4)	18.9 (17.3-22.8)	22.3 (17.9-24.0)	22.3 (19.4-25.6)	28.5 (24.0-30.9)	<0.001
DASH	71.0 (67.1-76.4)	49.9 (42.1-54.0)	40.2 (33.6-44.3)	31.0 (23.6-36.4)	26.2 (21.9-36.1)	<0.001

SPADI

**Figure 2:**
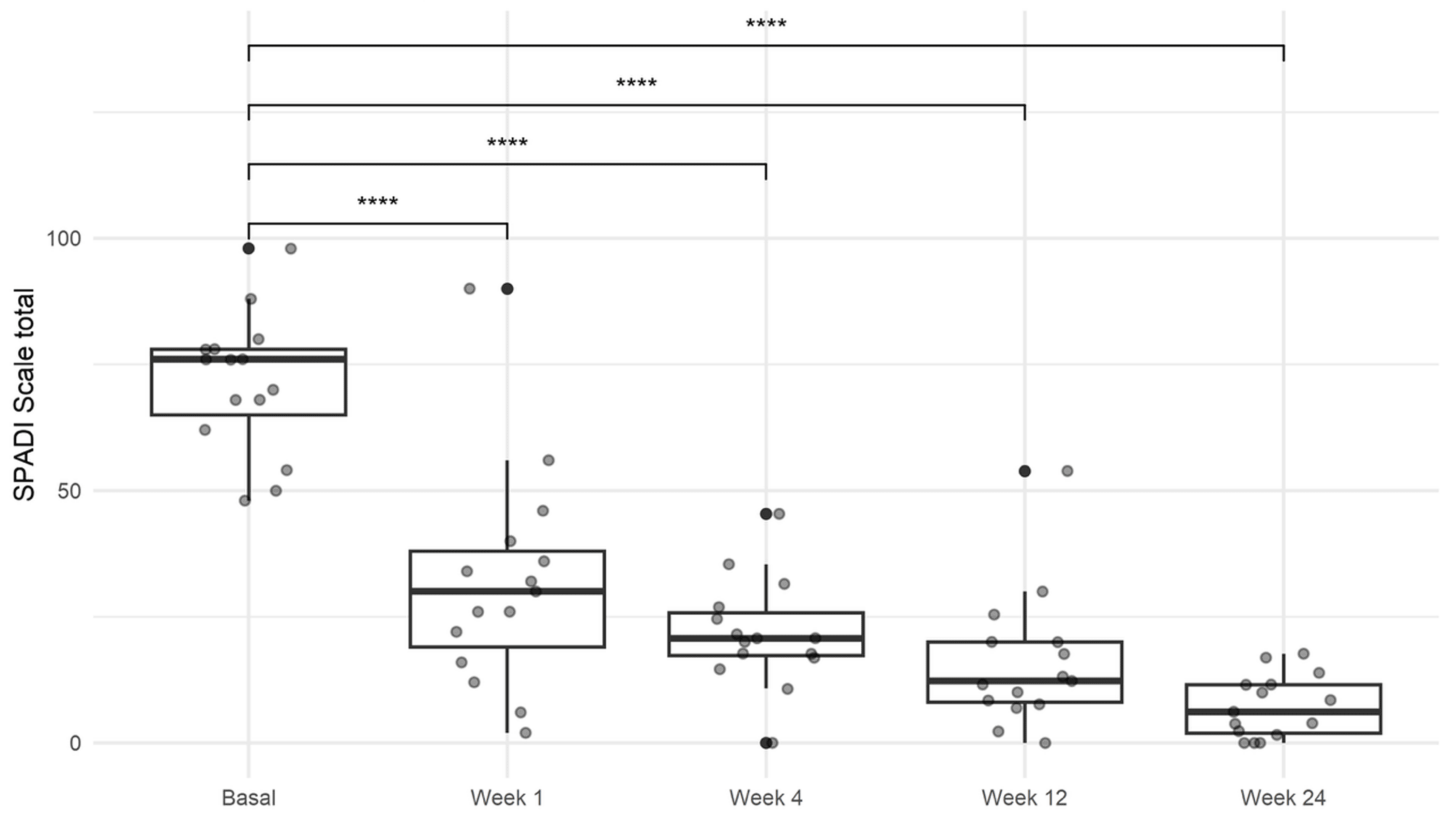
Results of the Shoulder Pain Disability Index (SPADI). A significant improvement in the score was sustained until week 24. One-way ANOVA with Tukey’s multiple comparison post hoc test. ****P < 0.0001. ANOVA, analysis of variance

The median baseline SPADI score was 76.1 (IQR 65.1-78.2). Subsequent assessments demonstrated a significant reduction in scores, with improvements maintained throughout follow-up. The final median score was 6.3 (IQR 1.8-11.7; *P *< 0.001; Figure 2; Table 2).

UCLA shoulder scale

**Figure 3:**
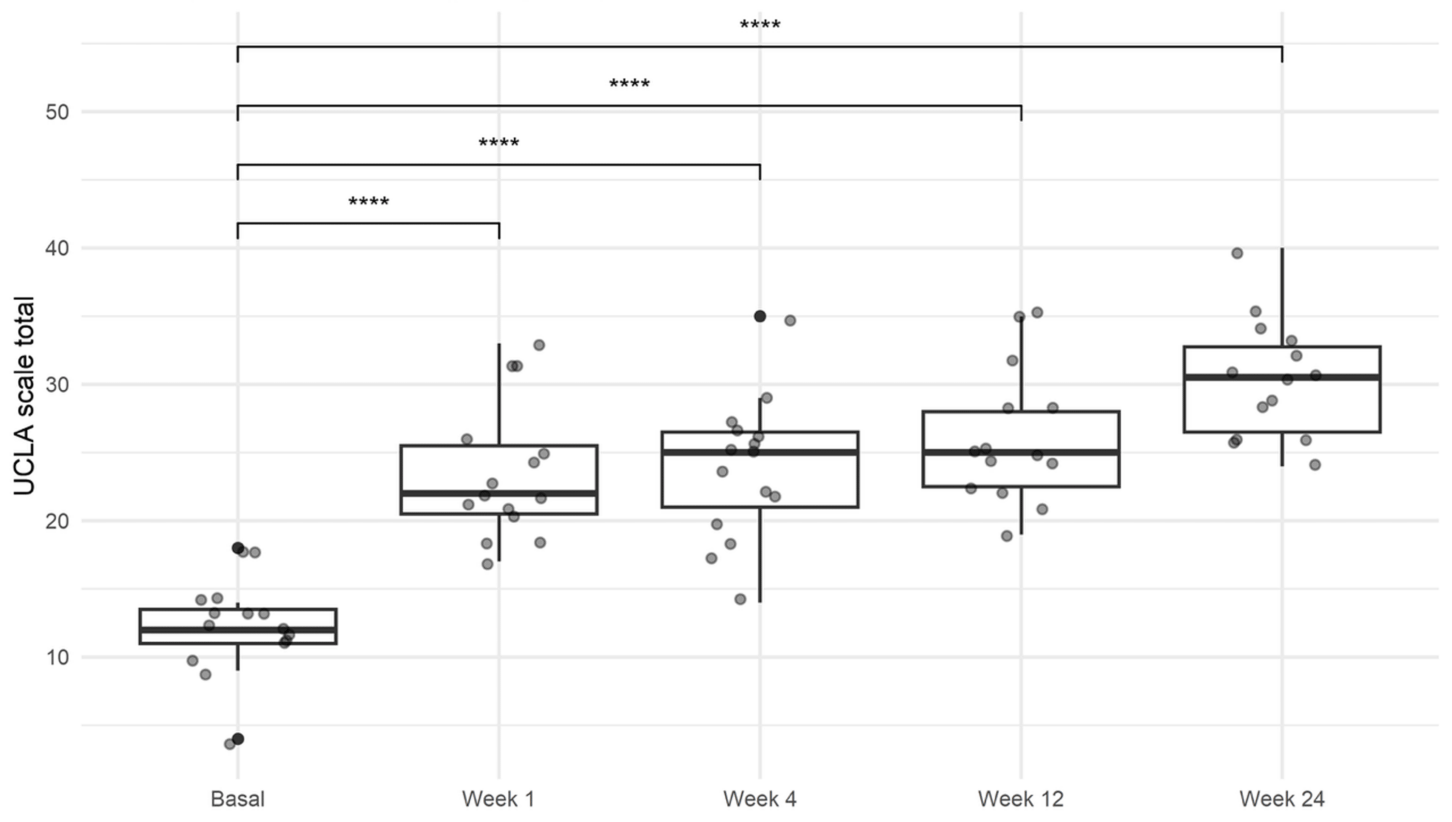
Results of the University of California at Los Angeles (UCLA) shoulder scale. An increase on the evaluation was observed during the follow-up of the patients. One-way ANOVA with Tukey’s multiple comparison post hoc test. ****P < 0.0001. ANOVA, analysis of variance

The initial median UCLA score was 7.7 (IQR 6.7-9.4). Scores progressively increased over the follow-up period, reaching a final median score of 28.5 (IQR 24.0-30.9), indicating good to excellent outcomes (*P *< 0.001; Figure 3; Table 2).

DASH score

**Figure 4:**
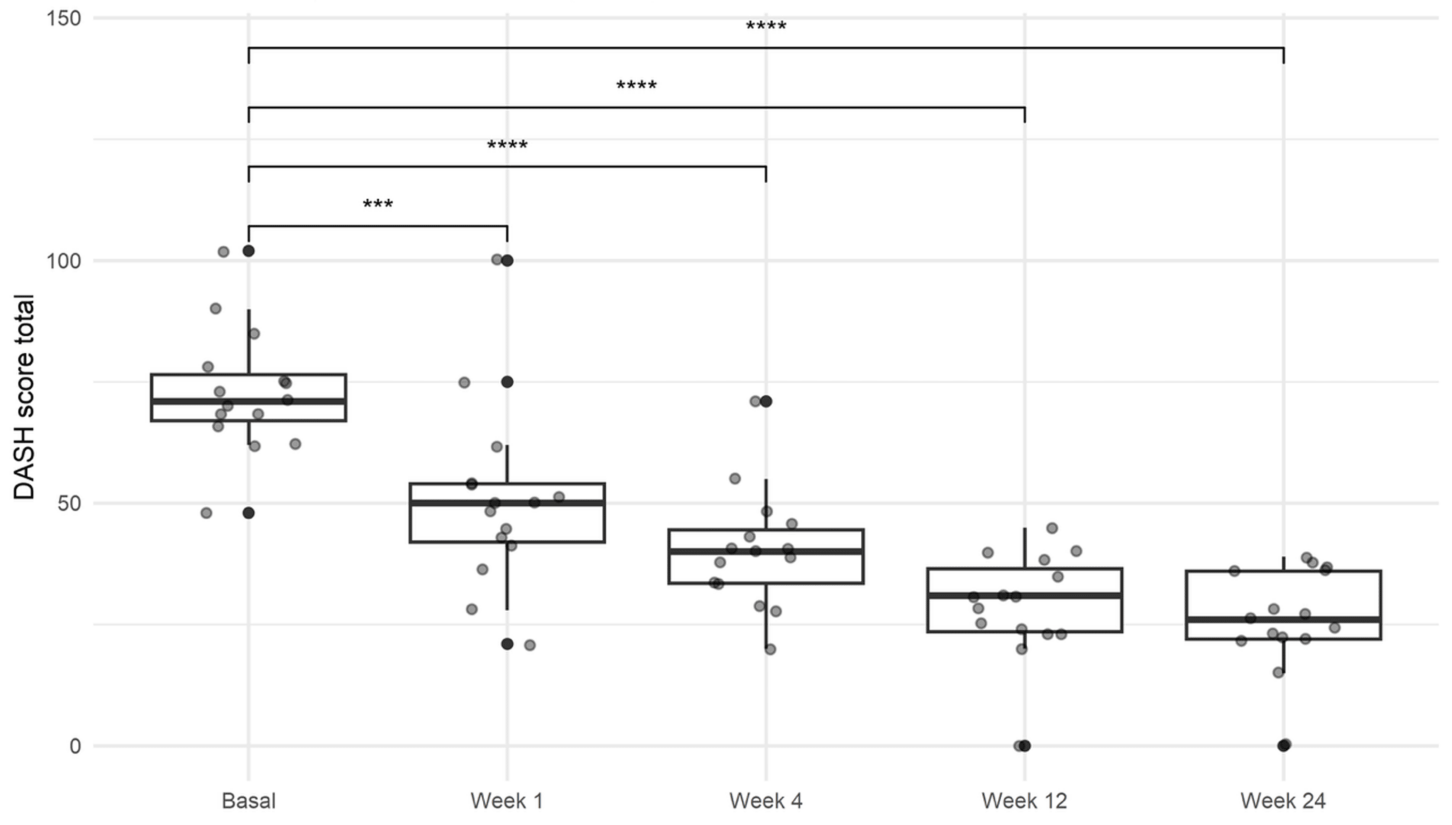
Results of the Disabilities of the Arm, Shoulder, and Hand (DASH) score. The improvement in disability and symptoms was recorded from week 1 to week 24. One-way ANOVA with Tukey’s multiple comparison post hoc test. ***P < 0.001. ****P < 0.0001. ANOVA, analysis of variance

The baseline median DASH score was 71.0 (IQR 67.1-76.4). A significant improvement was observed as early as the first-week evaluation, with a median score of 49.9 (IQR 42.1-54.0). This improvement persisted throughout follow-up, culminating in a final median score of 26.2 (IQR 21.9-36.1) (*P *< 0.001; Figure 4; Table 2).

Adverse events

No adverse effects were reported among the study participants.

## Discussion

Patients with painful shoulders caused by rotator cuff tendinopathy or glenohumeral arthritis can benefit from CRFA, demonstrating significant improvements in pain and clinical outcomes. Invasive radiofrequency (RF) treatment can be applied in two primary scenarios: as an adjunct following surgical procedures to facilitate early rehabilitation or as a viable alternative for patients who do not respond to conservative treatment and for whom surgery is not a suitable option.

RF treatment has been described as a promising minimally invasive procedure with advantages such as rapid recovery and a low incidence of adverse effects. This includes various RF modalities such as RFA, PRFA, and CRFA. Previous studies have primarily investigated RF treatments for knee joint pain. A meta-analysis has demonstrated that this therapy effectively reduces pain, although it does not significantly impact knee joint function. Furthermore, RFA has been reported to yield superior pain relief compared to PRFA [10].

According to Eckmann et al. [16], four primary target zones exist for radiofrequency ablation procedures: zone A, suprascapular branches; zone B, axillary ascending branches (posterior and anterior); zone C, lateral pectoral nerve; zone D, suprascapular branches and upper subscapular versus direct posterior cord branches. This standardized sequence of ablation was followed in our study.

Several studies have highlighted the benefits of RFA for chronic shoulder pain, including improvements in pain scores, extended pain relief duration, enhanced functional outcomes, and patient comfort with minimal complications [17-19]. Additionally, suprascapular nerve blocks have been widely used in multimodal analgesia for shoulder surgery. However, this effect is less pronounced in patients experiencing shoulder pain following non-shoulder surgeries, such as thoracotomy [20].

CRFA has demonstrated a pain relief rate of approximately 50% at six months and up to 74% in cases of knee joint pain [21-23]. Similar findings have been reported in shoulder OA, showing significant pain reduction and clinical improvement over six months of follow-up [11]. CRFA has also been successfully employed for various musculoskeletal conditions, including lumbar radiculopathy [24], knee OA, and post-total knee arthroplasty [25,26], yielding significant pain relief, increased duration of symptom relief, and improved quality of life.

The use of CRFA has demonstrated similar outcomes to MRFA in the treatment of chronic knee pain due to OA after one year of follow-up. The benefits of CRFA were observed beyond 24 weeks, with no significant differences at 52 weeks [27].

Steroid administration has been utilized following CRFA to mitigate post-neurotomy pain, with positive outcomes [28]. Based on these findings, corticosteroids were administered to all patients following the CRFA procedure in our study.

The minimal clinically important difference (MCID) represents the smallest measurable improvement in a score that patients perceive as beneficial [29]. The MCID for the UCLASS has been established at 3.5 points (range, 2.5-4.5) [30], while for the DASH, it is 10.2 points, and for the SPADI, it ranges from 8 to 13 points [25]. Our findings indicate that patients met the MCID thresholds for both the DASH and UCLA scales. Although the median SPADI improvement reached 6.1 points, all patients experienced notable clinical benefits.

This study has several limitations. It was a prospective cohort study without a comparative control group of patients with shoulder pain and a small number of included patients. Additionally, while RF treatment has been extensively studied for knee joints [23,24], fewer reports exist on its application for shoulder joint pain [11,17]. Our study included patients with diverse shoulder pathologies, some of whom had surgical indications; however, significant clinical improvement was observed across all participants throughout follow-up. The follow-up period was considered medium-term; however, a long-term follow-up of the patients would be beneficial to determine the true value of CRFA and identify any potential complications. Notably, no patient required surgical intervention for their primary shoulder pathology during the time of follow-up.

## Conclusions

CRFA is a minimally invasive medical procedure that can be considered to treat patients with shoulder pain of different etiologies. CRFA can be used as a co-adjuvant treatment to treat residual pain or in patients with surgical contraindications. The minimal clinically important difference of several shoulder scales can be obtained in patients treated with CRFA, reflecting an improvement of shoulder functionality and pain.
